# Delirious Mania in a 77-Year-Old Female

**DOI:** 10.7759/cureus.65619

**Published:** 2024-07-29

**Authors:** Eduardo D Espiridion, Ashley Deng, Lily Charron

**Affiliations:** 1 Psychiatry, Drexel University College of Medicine, Philadelphia, USA; 2 Psychiatry, Reading Hospital, West Reading, USA; 3 Psychiatry, Drexel University College of Medicine, West Reading, USA

**Keywords:** bells' mania, delirium mania, elderly, delirium, mania

## Abstract

Delirium is associated with acute episodes of disturbances in attention and awareness along with changes to cognition, including memory deficits and disorientation. Delirious mania (DM) is an unusual phenomenon where symptoms of delirium co-exist with symptoms of mania such as elevated or irritable mood, grandiosity, agitation, and cognitive disorganization. There is no formal agreement upon clinical symptoms for DM, but it generally includes acute onset of confusion, poor orientation, excitation, restlessness, and delusions. DM was first identified in the mid-1800s by Dr. Luther Bell and has only been identified by case reports since. We investigated a 77-year-old woman who was found at a gas station in an altered mental state. Upon observation, she has symptoms consistent with DM, including inappropriate laughter, distraction and confusion. She was diagnosed with acute metabolic encephalopathy, but the presentation of DM was considered in the differential and remains a unique finding.

## Introduction

Delirium is defined by the Diagnostic and Statistical Manual of Mental Disorders-Fifth Edition (DSM-V) as ‘A disturbance in attention (i.e., reduced ability to direct, focus, sustain, and shift attention) and awareness (reduced orientation to the environment)’. Delirious mania (DM) is an unusual phenomenon where, in conjunction with symptoms of delirium, symptoms of mania, including elevated or irritable mood, grandiosity, agitation, and cognitive disorganization co-exist. DM remains unclassified by the DSM-5 and International Classification of Diseases (ICD)-10 system. As such, it is difficult to determine the exact incidence of the disease [1]. 

There is no formal agreement upon clinical symptoms for DM, but generally, it can include acute onset of confusion, poor orientation, excitation, restlessness, and delusions. DM was formally identified in a report by Dr. Luther Bell in the mid-1800s. Some texts may use DM interchangeably with ‘Bell’s mania’. DM has only been identified by case reports [2]. Formal diagnoses of DM are complicated as many patients have pre-existing medical or neurological diseases or use psychoactive medications that can impact symptomology [3]. However, existing cases can be diagnosed via clinical presentation and the effectiveness of treatment. According to a chart review of 16 patients exhibiting both delirium and mania, ‘patients with delirium and mania had negative medical and neurological work-ups and were more likely to be younger, female, and with a prior diagnosis of bipolar disorder'. Sudden onset of symptoms, incontinence/inappropriate toileting, and denudativeness are distinctive features of the syndrome [3]. Yet another review of 14 cases exhibiting delirium and mania defined DM as a ‘syndrome of the acute onset of the excitement, grandiosity, emotional lability, delusions, and insomnia characteristic of mania, and the disorientation and altered consciousness characteristic of delirium'. Almost all patients exhibited signs of catatonia [2].

The relationship of DM is reportedly intertwined with catatonia, bipolar disorder, and mild schizophrenia [2]. As there are no currently established treatment guidelines, recognition of clinical symptomology is essential to treatment. Based on the findings of a case study, electroconvulsive therapy and high-dose benzodiazepines were deemed the most therapeutic treatments [3]. We report a case of a woman with no prior psychiatric history or concurrent psychoactive medication who presented with delusional and manic episode symptoms.

## Case presentation

History of present illness

The patient was a 77-year-old female who presented to the emergency department after being found at a local gas station in an altered mental state. Upon admission, the patient was found to be dehydrated and to have severe hypertension, with her blood pressure being as high as 227/92. The patient is currently retired and living alone. She reports being depressed due to her financial situation, blaming the government for her school taxes. 

She was easily distracted and incredibly tangential, making mental evaluation difficult. She was unable to remember why she was brought in. The patient appeared confused, disoriented, and disorganized with inappropriate loud laughter. She did not remember the date, where she was, what she ate last, or where she lived. She reported being on several psychiatric medications but could not name the dosages or who was prescribing them. She later denied taking any psychiatric medications at all. Upon psychiatric consultation, she appeared impulsive, paranoid, and with rapid shifts in mood and affect. Throughout conversation, she continued to laugh loudly and inappropriately. 

Past psychiatric history: Upon investigation it was found that she had been admitted to another hospital system four months ago for altered mental status. She was prescribed lithium and citalopram though her blood work returned a lithium level of 0, indicating non-adherence. She denied prior psychiatric hospitalizations, hallucinations, or suicidal or homicidal ideation. She has a medical history of bipolar disorder and cognitive decline.

Laboratory and imaging

**Figure 1:**
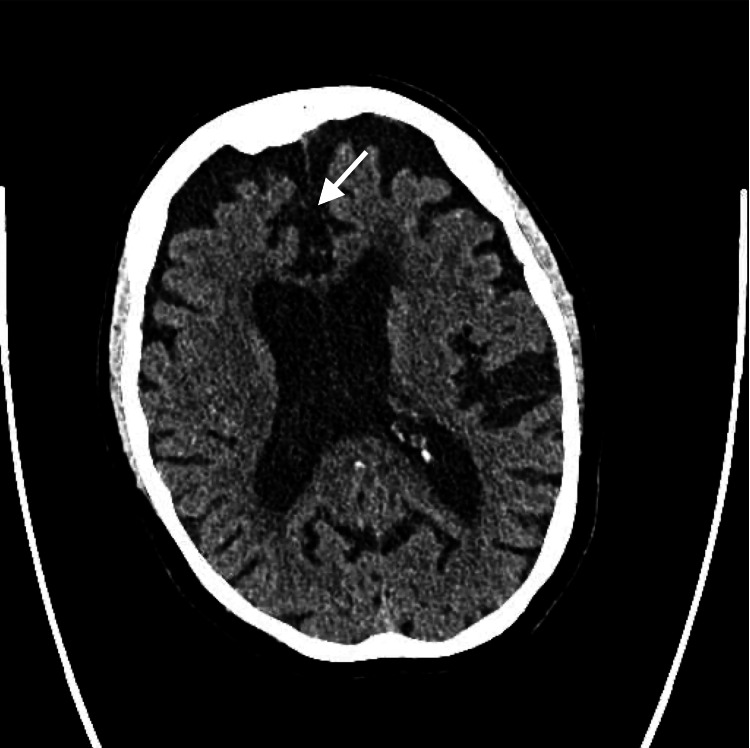
CT scan of the head The image shows cortical atrophy (white arrow).

**Figure 2:**
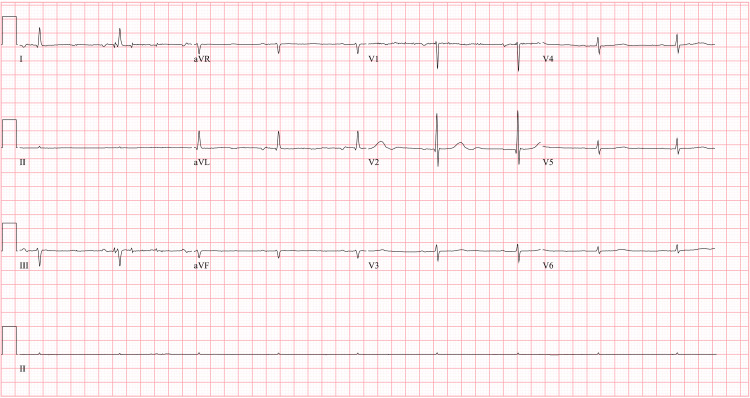
EKG of the patient at the time of admission EKG shows sinus bradycardia and non-specific T-wave abnormality.

The CT of her brain was negative for acute abnormality. The patient’s brain CT shows cortical atrophy consistent with her age (Figure 1). The CT also showed suspected chronic microvascular ischemic gliosis and an old lacunar infarct at the right basal ganglia. Her urinalysis had traces of bacteria, WBCs, positive leukocyte esterase, and a cloudy appearance (Table 1). She was unable to provide clinical history, so the possible UTI was treated empirically with fosfomycin. Her ECG was typical for her age and cardiovascular history (Figure 2).

**Table 1:** Relevant lab values from urinalysis lab: Laboratory examination.

Component	Result	Reference Range
Ketones	Trace	Negative
Blood	Trace	Negative
Protein	30 mg/dL	Negative
Leukocytes	Moderate	Negative
Bacteria	Occasional	Negative
WBC	11-20 WBC/HPF	0-5
Benzodiazepines	Negative	Negative

Treatment

On day one, following the initial workup, the patient was started on nicardipine infusion therapy to lower blood pressure. The patient’s blood pressure stabilized. She was also started on olanzapine tablets. Her mental state, including concentration, mood swings, and confusion rapidly improved following administration of medications, though inappropriate laughter remained. 

Metabolic encephalopathy and uncontrolled bipolar disorder were considered. She was ultimately discharged with a diagnosis of cognitive disorder. She was prescribed atorvastatin and lisinopril to manage hypertension. She was restarted on citalopram and olanzapine to address the bipolar disorder. After four days, the patient was discharged. The patient gave verbal and written consent to the provider for case report.

## Discussion

Diagnostic challenges

The presentation of this patient’s symptoms is distinctive in that it showcases a complex interplay between delirium and hypomania, with additional factors complicating the clinical picture. The patient's easy distractibility and fluctuations in concentration are hallmark features of delirium, a state characterized by acute confusion and altered consciousness. However, the uniqueness of this case lies in the co-occurrence of hypomanic-like behavior, which includes inappropriate laughter, increased volume of speech, agitation, and an inability to maintain factual consistency about her medical history. These symptoms deviate from the typical presentation of delirium and suggest an underlying manic or hypomanic episode.

Additionally, the CT scan showed mild cortical atrophy. However, slight cortical atrophy in the elderly can be physiological [4]. There was no evidence of subcortical atrophy or atrophy in regions normally associated with delusions like the frontal and temporal cortex [5]. The blood and urine samples did not show any psychoactive substances. The patient’s age adds another layer of complication in diagnosis and treatment, as certain medications are found to be more or less effective when considering elderly delirium. 

The consideration of delirium secondary to a urinary tract infection (UTI) was a reasonable one, given that UTIs are a known cause of delirium, especially in older adults. However, the UTI was not confirmed and treated empirically. Based on her clinical history and presentation of symptoms, the UTI did not appear to be a likely cause. When considering differentials, uncontrolled bipolar disorder and hypertensive encephalopathy predominated.

Treatment 

The patient's presentation was further complicated by severe hypertension, which was identified as a significant contributing factor. The diagnosis of acute metabolic encephalopathy was made after observing marked improvement in behavior following the administration of IV nicardipine (a medication used to manage hypertension). This improvement highlights the critical role of hypertension management in resolving encephalopathic symptoms.

Though benzodiazepine treatment may have been therapeutic for managing bipolar disorder following discharge, benzodiazepine has been associated with worsening delirium in the elderly due to pharmacological and physiological effects [6]. As a result, the patient was prescribed olanzapine, an atypical antipsychotic with a lower adverse effect profile compared to typical antipsychotics and benzodiazepines [7]. The patient's mental state rapidly improved two days after administration of olanzapine and nicardipine. This rapid reversal supports the diagnosis of metabolic encephalopathy but also the symptomology of DM, as true bipolar disorder typically takes days to weeks to show improvement following antipsychotic administration [8]. 

Clinical implications

Classification of DM remains ambiguous. Historically, DM has been classified within or associated with bipolar disorder, catatonia, and schizophrenia. In some previous studies, DM was classified as a type of bipolar disorder [9], a type of manic disorder. Though there is overlap in catatonia and bipolar disorder with DM, there are also distinctions. For example, the sudden onset of DM occurs over hours to days versus the longer time course of at least a week seen in bipolar disorder. Similarly, DM can present with or without catatonic features [10]. Though some cases of DM occur secondary to diseases including bipolar disorder and schizophrenia, many occur in insolation. This indicates the potential necessity to create a formalized classification for DM. 

The lack of a distinct classification makes treatment of DM especially complicated. In several case-studies, traditional treatment for mania, lithium, was shown to be effective in DM remission [11-12]. DM is also frequently associated with catatonia, which was once a subtype of schizophrenia but has since become its own syndrome due to the prevalence of catatonia not associated with schizophrenia [2]. Electroconvulsive therapy (ECT), normally used to treat catatonia, has been identified by several case studies as an especially effective treatment for DM remission [3,9]. Additionally, high-dose benzodiazepines, such as lorazepam, can be effective in reducing DM symptoms [3]. Our patient had no history of ECT and a completely negative urine and blood sample for various drugs including benzodiazepine and lithium level, suggesting non-adherence.

## Conclusions

DM is an unusual disease that may not be uncommon and can have lethal consequences if not treated. The lack of distinct categorization and treatment guidelines makes DM difficult to diagnose and treat. However, symptomatology recognition and medical history can help, as in the case of this patient with a history of bipolar disorder and presenting with symptoms of delirium and mania including easy distractibility, inappropriate laughter, and mood swings. The ultimate diagnosis of ‘cognitive disorder’ reflects the multitude of problems affecting her cognition including metabolic encephalopathy due to severe hypertension, uncontrolled bipolar disorder, and presentation of DM symptoms. The treatment plan focused on addressing these contributing factors. This case adds to the limited body of literature surrounding this phenomenon. Whether DM is primary or secondary to the disease, we emphasize the importance of clinical recognition and diagnosis, as DM appears to be treatable. As most of the literature surrounding DM is case-based, future studies should become more rigorous, including prospective and retrospective cohort studies.

## References

[1] Klerman GL (1981). The spectrum of mania. Comprehensive Psychiatry.

[2] Fink M (1999). Delirious mania. Bipolar Disord.

[3] Karmacharya R, England ML, Ongür D (2008). Delirious mania: clinical features and treatment response. J Affect Disord.

[4] Fjell AM, Walhovd KB, Fennema-Notestine C (2009). One-year brain atrophy evident in healthy aging. J Neurosci.

[5] Sultzer DL, Leskin LP, Melrose RJ, Harwood DG, Narvaez TA, Ando TK, Mandelkern MA (2014). Neurobiology of delusions, memory, and insight in Alzheimer disease. Am J Geriatr Psychiatry.

[6] Ettcheto M, Olloquequi J, Sánchez-López E (2019). Benzodiazepines and related drugs as a risk factor in Alzheimer’s disease dementia. Front Aging Neurosci.

[7] Maguire GA (2000). Impact of antipsychotics on geriatric patients: efficacy, dosing, and compliance. Prim Care Companion J Clin Psychiatry.

[8] Vieta E, Sanchez-Moreno J (2008). Acute and long-term treatment of mania. Dialogues Clin Neurosci.

[9] Lee BS, Huang SS, Hsu WY, Chiu NY (2012). Clinical features of delirious mania: a series of five cases and a brief literature review. BMC Psychiatry.

[10] Pereira Herrera M, Zimmerman AM (2021). Case of refractory delirious mania responsive to lithium. BJPsych Open.

[11] Bond TC (1980). Recognition of acute delirious mania. Arch Gen Psychiatry.

[12] Jacobowski NL, Heckers S, Bobo WV (2013). Delirious mania: detection, diagnosis, and clinical management in the acute setting. J Psychiatr Pract.

